# Boosting the cell voltage in biphasic flow batteries *via* Galvani potential difference†

**DOI:** 10.1039/d4cp01402b

**Published:** 2024-06-05

**Authors:** Vahid Abbasi, Pekka Peljo

**Affiliations:** a Research Group of Battery Materials and Technologies, Department of Mechanical and Materials Engineering, Faculty of Technology, University of Turku 20014 Turku Finland pekka.peljo@utu.fi

## Abstract

Galvani potential differences between aqueous and organic phases of biphasic flow batteries can be utilized to boost the cell voltage by *ca.* 600 mV. This effect is demonstrated by comparing batteries utilizing three different solvents, trifluorotoluene, dichloroethane and propylene carbonate, with ferrocene and decamethyl ferrocene as model organic redox couples.

## Abstract 2

Galvani potential differences between aqueous and organic phases can increase the cell voltage of biphasic flow batteries by 600 mV.

## Graphical abstract

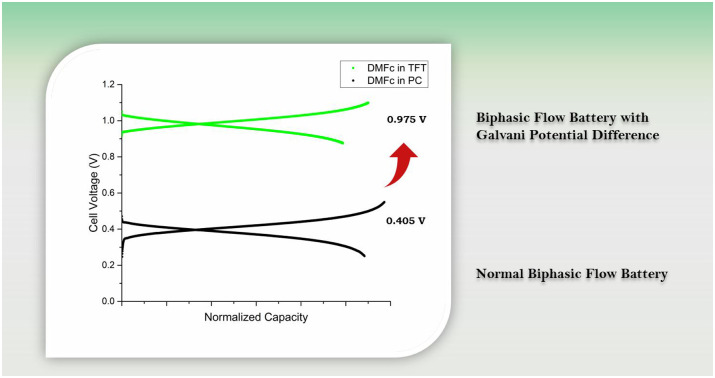

In conventional flow batteries, the fundamental principle involves the utilization of two redox couples dissolved in solvents, allowing for the storage and release of electrical energy through the process of charge and discharge.^1^ A comprehensive overview of the technology is given in a recent book,^2^ and more specific developments of the chemistries are described in some recent reviews.^3,4^ The evolving energy storage landscape has led to the emergence of biphasic flow batteries, employing two immiscible electrolytes, which utilize organic solvents for a higher cell voltage compared to water-based batteries.^5–8^

Unlike their monophasic counterparts, biphasic flow batteries introduce a novel approach by employing two immiscible liquid phases (immiscible negolyte and posolyte), often a combination of organic and aqueous phases,^5–8^ two organic phases separated by an aqueous phase^9,10^ or more recently also two aqueous-containing phases.^6^ Recently, also all-organic biphasic batteries have been proposed.^11^ The cell voltage in flow batteries is defined as the difference of the redox potentials of the positive and negative redox couples: *E*_cell_ = *E*_pos_ − *E*_neg_.

It has been well known since the 1970s that the interface between immiscible electrolyte solutions (ITIES) can be significantly polarized, resulting in Galvani potential differences of up to 0.7 V between phases.^12–15^ For thermodynamic definition of the Galvani potential difference, see the ESI.† We have shown earlier that the redox potential difference between two redox couples is influenced by the Galvani potential difference between the two phases.^10,16^ Therefore, the cell voltage in biphasic systems can be expressed as *E*_cell_ = *E*_pos_ − *E*_neg_ + Δ*ϕ*, where Δ*ϕ* is the Galvani potential difference of the phase containing the positive couple and phase containing the negative couple. This equation is also valid in the case of Galvani potential differences arising from, for example, liquid junction potentials formed when two electrolytes of different concentration are in contact,^17^ or Donnan potentials over membranes arising from unequal concentrations of ions between the two electrolytes.^17^ For example, the Donnan potential has been shown to have a *ca.* 40 mV increasing effect on the cell voltage of vanadium flow batteries.^18^ The key difference between the Galvani potential difference generated in biphasic systems and by liquid junction or Donnan potentials is that the potential difference for the latter two includes only the concentration difference of the two electrolytes *c*_1_ and *c*_2_.^17^1
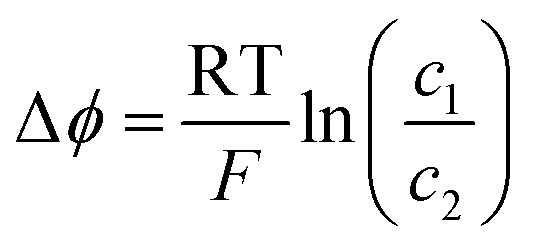
while for biphasic systems the additional term Δ*ϕ*_*ι*_^0^ is included,^12–15^ arising from the difference of solvation energy of species *i* between the two phases.2
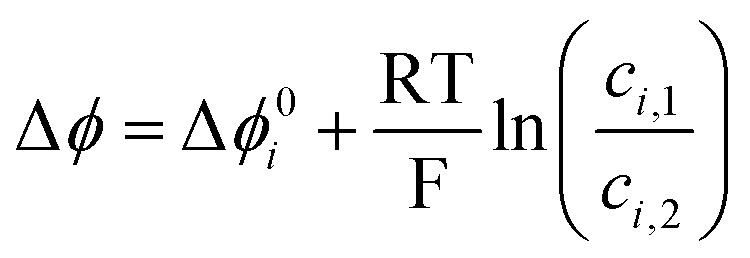


Surprisingly, no biphasic flow battery taking advantage of this additional potential has been reported, although our earlier work demonstrated that an oil–water–oil system taking advantage of two Galvani potential differences could in essence store energy by transferring a salt from aqueous to oil phases.^10^ In this work we demonstrate that Galvani potential difference can also be utilized to significantly increase the cell voltage of biphasic flow batteries.

Liquid–liquid interfaces can be polarized electrochemically with an external power supply, but also chemically by partition of a common ion.^12–15^ The degree of immiscibility between the two solvents is proportional to the generated Galvani potential difference by partition of salts.^19,20^ The greater the difference in immiscibility between the organic and aqueous phases, the more pronounced the Galvani potential difference becomes with the same salt, as recently discussed in detail by Samec *et al.*^19,20^ This property opens up exciting possibilities for optimizing the performance of biphasic flow batteries, as the tailored selection of solvents and their immiscibility can be strategically engineered to enhance the overall voltage and energy output of the system.

In this study, polarization of the aqueous interface with trifluorotoluene (TFT), dichloroethane (DCE) and propylene carbonate (PC) as solvents of a biphasic flow battery (Fig. 1) was explored. The miscibility of these solvents with water is shown in Table 1.

**Fig. 1:**
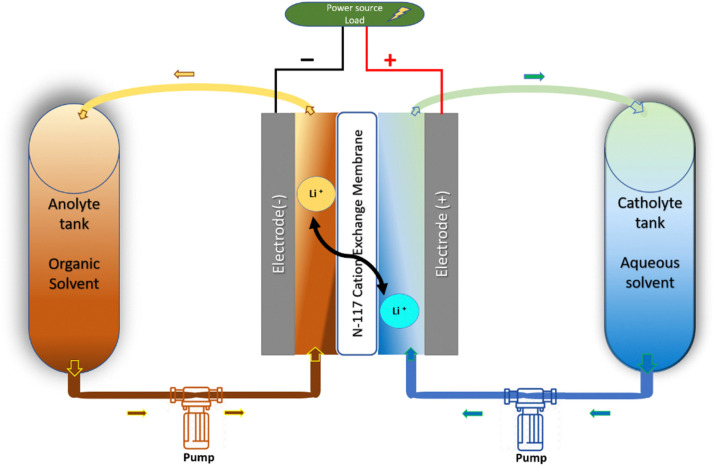
Schematic of a biphasic flow battery indicating the Galvani potential difference of Li^+^.

**Table 1:** Miscibility organic solvent–water systems

Organic solvent	Solubility of solvent in water^a^ (wt%)	Solubility of water in solvent^a^ (wt%)
Trifluorotoluene (TFT)	<0.1	0.04
Dichloroethane (DCE)	0.87	0.16
Propylene carbonate (PC)	17.5	8.30

a Ref. 19–24.

The thermodynamics of partition of ions at the interface of two immiscible electrolytes (ITIES) is well understood.^12–15^ As measurements between pure solutions would be difficult, all the available data focuses on mutually saturated solutions. The Galvani potential difference of the interface depends on the partitioning of different ions, and can be evaluated when the Gibbs energy of transfers of all the charged species from aqueous to organic phases are known. In this work, the interface between two phases is polarized by the addition of lithium tetrakis(pentafluorophenyl)borate salt (LiTB), leading to partitioning of the salt between the two phases. As the Gibbs energies of transfer of all charged species have been measured or can be estimated as listed in the ESI,† the equilibrium Galvani potential differences for TFT and DCE with water are estimated theoretically as 0.734 V and 0.519 V, respectively, as described in the ESI,†.^25,26^ Meanwhile the Galvani potential difference for water and PC is approximately 0 V.^21^ The thermodynamic analysis shows that for the DCE the potential difference arises from partitioning of Li^+^ in DCE, and both the Li^+^ and TB^−^ for TFT. The origin of the differences has been proposed to result from solvation and partial removal of the hydration shell of Li^+^ in TFT.^19,20^ It seems that the Galvani potential difference obtained by the partition of lithium is limited to *ca.* 0.7 V as it is challenging to find solvents that would have even lower solubility of water than TFT but still have enough polarity to allow sufficient solvation of salts to allow electrical conductivity.

In the construction of a biphasic flow battery, one redox couple is required for each phase. For the aqueous phase, a redox electrolyte consisting of 30 mM of both potassium hexacyanoferrate (ii) and potassium hexacyanoferrate (iii) (referred to as KFCN) in excess, with 100 mM lithium chloride as the supporting electrolyte, was chosen as a typical model system. For the organic phase, two typical redox couples ferrocene (Fc) and decamethylferrocene (DMFc) were utilized along with 10 mM lithium tetrakis(pentafluorophenyl)borate (LiTB) to polarize the interface. Detailed information on the experimental details of all the biphasic batteries assembled can be found in Table S1 (ESI†). In brief, a filter press-type flow cell placed inside the glove box was used in the flow-through configuration utilizing carbon felt electrodes and a Nafion 117 membrane to separate the phases. The organic solvent is saturated with water as water can pass through the Nafion 117 membrane.

The cell voltage can be estimated by eqn (3).^10,16^3
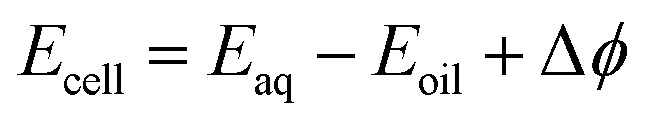
when redox potentials of aqueous and organic redox couples as well as the Galvani potential difference are known. Galvani potential differences were estimated in the ESI,† while the standard potentials of Fc and DMFc *vs.* standard hydrogen electrode (SHE) in TFT^16,27,28^ and DCE,^29–31^ and KFCN in water^16,32^ are known. The standard potential of Fc in PC can be estimated based on the literature data^33,34^ and the potential for DMFc was measured in this work (see ESI†). Using the known potentials and the KFCN potential *vs.* SHE (0.358 V reported by Vanysek^32^ or 0.467 V in 100 mM LiCl by Smirnov^16^), the battery potential can be estimated with eqn 3.^13^ All the potentials are tabulated in Table 2, utilising the aqueous formal potential evaluated in ref. 16.

**Table 2:** Redox potential of the couple in the organic phase, estimated Galvani potential difference, theoretical cell voltage calculated from eqn 3 as well as the measured cell voltage

Oil phase	*E* ^0^ in oil phase *vs.* SHE (V)^a^	Estimated Galvani potential difference (V)	Theoretical voltage (V)	Measured voltage (V)
DMFc in TFT	0.08	0.734	1.121	0.975
DMFc in DCE	0.07	0.519	0.916	0.852
DMFc in PC	0.055^b^	≈0	0.412	0.405
Fc in TFT	0.720; or 0.736	0.734	0.481	0.361
Fc in DCE	0.640	0.519	0.346	0.239
Fc in PC	0.576	≈0	−0.109	−0.135

a Ref. 16 and 27–34.

b Estimated from cell potential from Fc potential (ESI). Estimated Galvani potential difference means the Galvani potential difference calculated by solving equation S17. Theoretical voltage means the voltage we expect from eqn 3, and measured voltage is the voltage we get from experiments, Fig. 2.

To demonstrate that Galvani potential difference Δ*ϕ* can indeed have a positive effect on the cell voltage, charge and discharge cycling was performed with the flow batteries. As tests were performed with different DMFc and Fc concentrations and volumes, the charge and discharge curves for each case are shown as cell voltage *vs.* capacity in Fig. 2 and Fig. S7 (ESI†) while the measured voltage at *ca.* 50% state of charge is reported as measured voltage in Table 2. The measured potentials also agree rather well with the expected theoretical values.

**Fig. 2:**
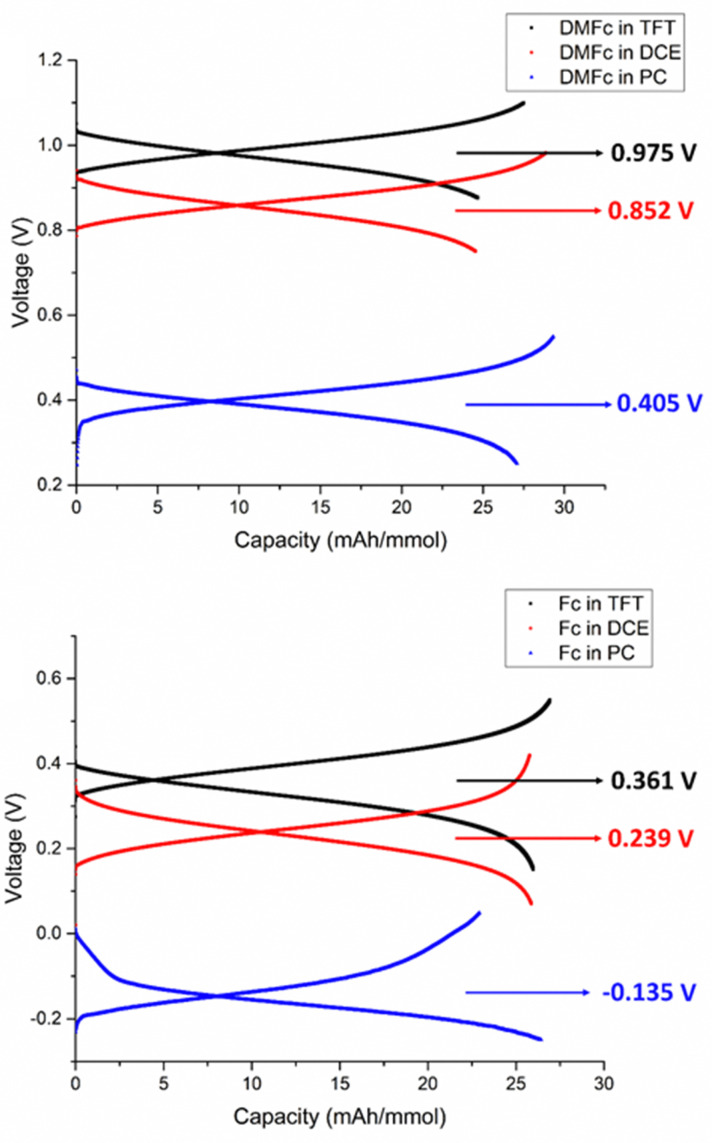
Charge and discharge profiles from battery experiments. top) Comparing decamethylferrocene (DMFc) in trifluorotoluene (TFT), dichloroethane (DCE), and propylene carbonate (PC). bottom) Comparing ferrocene (Fc) in the same solvents.

As the aqueous redox couple is in excess compared to DMFc or Fc in the organic phase and the original LiTB concentration in the organic phase was 4–10 times higher than the DMFc or Fc concentration, charge was limited by conversion of DMFc or Fc to the oxidized form. The experiments show that the polarization of the cell is not significant. This is because low current densities of <200 mA cm^−2^ were used, resulting in small *iR* drop of <150 mV as shown in the ESI.† Average potential close to 50% state of charge was extracted from the experiments, and comparison of the differences between measured values from the battery correspond well with the differences in the standard potentials of DMFc and Fc. The cell voltage difference between DMFc and Fc in TFT is 0.614 V, while the expected value would be 0.640 V. The corresponding values for DCE are 0.613 V *vs.* 0.570 V (see ESI†).

The cell voltages based on eqn 3 are 100–150 mV higher for TFT, 60–100 mV higher for DCE and 10–50 mV higher for PC than the actual measured values. There might be some error in the method to evaluate the standard potential of Fc in PC or KFCN in 100 mM LiCl. Additionally, this difference could originate from the membrane potential over Nafion,^17^ or from non-ideal activity coefficients especially in the organic solvents. The effect of the activities was estimated as *ca.* −45 mV for TFT, −37 mV for DCE and no significant effect for PC considering the extended Debye–Hückel equation for non-aqueous solutions (see ESI†), improving the agreement with theoretical and measured voltages (see ESI†). This indicates that the membrane contribution would be 30–100 mV. As the standard potentials of DMFc and Fc do not significantly change between each solvent, Fig. 2 clearly shows that the cell voltage of biphasic flow batteries can be boosted by almost 600–700 mV by choosing appropriate solvents and polarizing salts. Improved cell voltages could be obtained by replacing Fc or DMFc with more negative redox couples.

The continuous cycling of batteries shown in Fig. S8–S10 (ESI†) for more than 10 cycles illustrates that stable cycling can be achieved and the cell voltage remains constant during cycling. However, the cycling performance of the batteries and long-term cycling stability suffered from the evaporation of the organic solvents. When electrochemical reactions occur, an ion must carry the charge across the liquid–liquid interface to upkeep the electroneutrality. In this case the charge needs to be carried also across the Nafion membrane, so Li^+^ emerges as the predominant species responsible for charge transport. The kinetics of this ion transfer reaction can be described with the Butler–Volmer formalism, with an apparent standard rate constant of *ca.* 0.1–0.4 cm s^−1^ measured for a tetraethyl ammonium ion.^35^ As this reaction can be considered very facile, no significant polarization losses are expected due to the ion transfer reaction at the liquid–liquid interface. However, higher resistance of the system (see ESI† for further details) compared to water-based flow batteries is a disadvantage. For example, the resistance from *iR* drop varied between 10–150 mV already at very low current densities.

Additionally, as the battery is charged, Li^+^ is transferred into the aqueous phase. Therefore, the amount of LiTB electrolyte in the organic phase has to initially be higher than the amount of the redox species to avoid the loss of the polarization of the interface induced by partitioning of Li^+^ in the two phases. These disadvantages could be overcome with microemulsion-based systems employing low volatility organic solvents, if ionic conduction could be realized in the less resistive aqueous phase.^36,37^ Exploring various alternative membranes to achieve solution separation without relying on Nafion, we found that the exclusive use of Nafion in the flow battery cell prevented solution mixing (more details in the ESI†).

This significant enhancement in cell voltage demonstrates the potential for developing biphasic flow batteries that outperform conventional systems and lead to more efficient and advanced energy storage solutions.

## Conclusions

Three immiscible organic solvents with different solubility in water have been tested as solvents for biphasic flow batteries. The lower the solubility of the organic solvent in water, the higher the Galvani potential difference. We demonstrate that the Galvani potential difference can boost the cell voltage of biphasic flow batteries by up to 600–700 mV. The low conductivity of organic solvents compared to water, their low boiling points, and their incompatibility with general cell materials are drawbacks that can make working with them more challenging.

This significant enhancement in cell voltage demonstrates the potential for developing biphasic flow batteries that outperform conventional systems and lead to more efficient and advanced energy storage solutions. The concept could be exploited for increasing the voltage of biphasic battery systems reported in the literature, and for example in microemulsion based biphasic batteries. However, the drawbacks of the system need to be overcome to result in performance on par with the state-of-the-art flow batteries.

## Author contributions

V. A: conceptualization, formal analysis, investigation, methodology, validation, visualization, writing – original draft, writing – review and editing. P. P: conceptualization, funding acquisition, resources, supervision, writing – original draft, writing – review.

## Conflicts of interest

There are no conflicts to declare.

## Supplementary Material

CP-026-D4CP01402B-s001
