# Supplementary material for: Boosting the cell voltage in biphasic flow batteries via Galvani potential difference
Source: Phys Chem Chem Phys. 2024 Jun 5;26(25):17476–80. doi: 10.1039/d4cp01402b (PMC11202308; doi:10.1039/d4cp01402b)
Supplement: CP-026-D4CP01402B-s001 [file CP-026-D4CP01402B-s001.pdf]

Electronic Supplementary Material (ESI) for Physical Chemistry Chemical Physics.
This journal is © the Owner Societies 2024

Supplementary Information

Boosting the Cell Voltage in Biphasic Flow Batteries via Galvani Potential Difference

Vahid Abbasi,[a] Pekka Peljo [a]*

Research Group of Battery Materials and Technologies, Department of Mechanical and Materials Engineering, Faculty of Technology, University of Turku, 20014 Turku, Finland.

- Corresponding author, e-mail: pekka.peljo@utu.fi

Contents

Chemicals ... 2
Safety & hazards ... 2
Electrochemical experiments ... 3
Membrane testing ... 4
Battery testing ... 6
Estimation of the standard potentials of Decamethylferrocene and Ferrocene in Polypropylene Carbonate ... 6
Reactions in the cell ... 7
Thermodynamics of the ITIES and partitioning of the species ... 8
Battery experiments ... 13
Cyclability and coulombic efficiency of batteries ... 16
Cyclic Voltammograms ... 20
Impedance spectra ... 21
Supplementary References ... 22

Chemicals

All chemicals were used without further purification. All aqueous solutions were prepared with ultra-pure water (Millipore Direct-Q® 5 UV, 18.2 MΩ·cm). Decamethylferrocene (DMFc 97 %), Lithium chloride (LiCl +99 %), Propylene carbonate (PC 99.7 %), and 1,2-dichloroethane (DCE +99 %) were purchased from Sigma Aldrich. Potassium hexacyanoferrate (II) +99 % and Potassium hexacyanoferrate (III) +99 % were purchased from VWR. α,α,α-trifluorotoluene (TFT +99 %) was purchased from Thermoscientific. Ferrocene (Fc +99 %) was purchased from Alfa Aesar. Lithium tetrakis(pentafluorophenyl)borate ethyl etherate (LiTB) was purchased from Boulder Scientific. Nafion ™ N-117 Membrane was purchased from Ion-power. Thermally activated carbon felts GFD 4.6 mm from SIGRACELL as electrode for battery tests.

Safety & hazards

Caution: According to the material safety data sheet 1,2-dichloroethane is classified as toxic and possibly carcinogenic. It has high vapour pressure, and it is highly flammable.

Handling: Wear personal protective equipment/face protection. Do not get in eyes, on skin, or on clothing. Do not ingest. If swallowed then seek immediate medical assistance. Use only under a chemical fume hood. Do not breathe mist/vapors/spray. Keep away from open flames, hot surfaces and sources of ignition. Use only non-sparking tools. To avoid ignition of vapors by static electricity discharge, all metal parts of the equipment must be grounded. Take precautionary measures against static discharges.

Storage. Keep containers tightly closed in a dry, cool and well-ventilated place. Keep away from heat, sparks and flame. Incompatible Materials. Strong oxidizing agents. Bases. Alkali metals.

According to the material safety data sheet α,α,α-trifluorotoluene is highly flammable liquid and vapor and toxic to aquatic life with long lasting effects. Similar precautions should be employed as working with DCE.

Electrochemical experiments

Cyclic voltammograms (CVs) were recorded after 10 minutes N_2 bubbling using a SP-240 (BioLogic, France) potentiostat at room temperature (*ca*. 20-22 °C). Three electrode experiments were performed with glassy carbon electrode (radius 1 mm), Ag/Ag^+ reference (10 mM $AgNO_3$ in acetonitrile) and a platinum counter electrode. Battery Cycler G340A (LANHE, China) and Pump BT600M (Baoding Chuangrui, China) were used for battery tests.

The tests were performed in a Mbraun glovebox under nitrogen (N_2) atmosphere at room temperature (*ca*. 25 °C) in flow battery cell made of Polypropylene and Solve-Flex tubing from MasterFlex compatible with the solvents in use, were employed for the experiments (see Fig. S1). The flow rate of 30 mL min^{-1} set for all experiments.

In a redox flow battery cell, the assembly starts with endplates which provide structural support and ensure even distribution of pressure. Attached to these endplates are current collectors made of graphite sheets to collect the electrons from reactions happening in carbon felts, these felts, known for their porous nature and high surface area, are integral for effective chemical reactions. An ion-selective membrane is placed between the compartments containing the carbon felts (2 x 2.5 cm), allowing for the transfer of ions while keeping the electrolyte solutions separate. The area of the membrane in contact with the electrolytes is 5 cm^2. The current density is determined by dividing the current by the membrane area, which in this case is 2 x 2.5 cm, equal to the surface area of the carbon felt electrode. Various current densities were examined to ascertain that any resistance encountered

originates from the solution rather than the passage of Li^+ ions across the interface. Ensuring the integrity of the cell, gaskets and seals are positioned around the edges to prevent leakage and cross-contamination of the electrolytes.

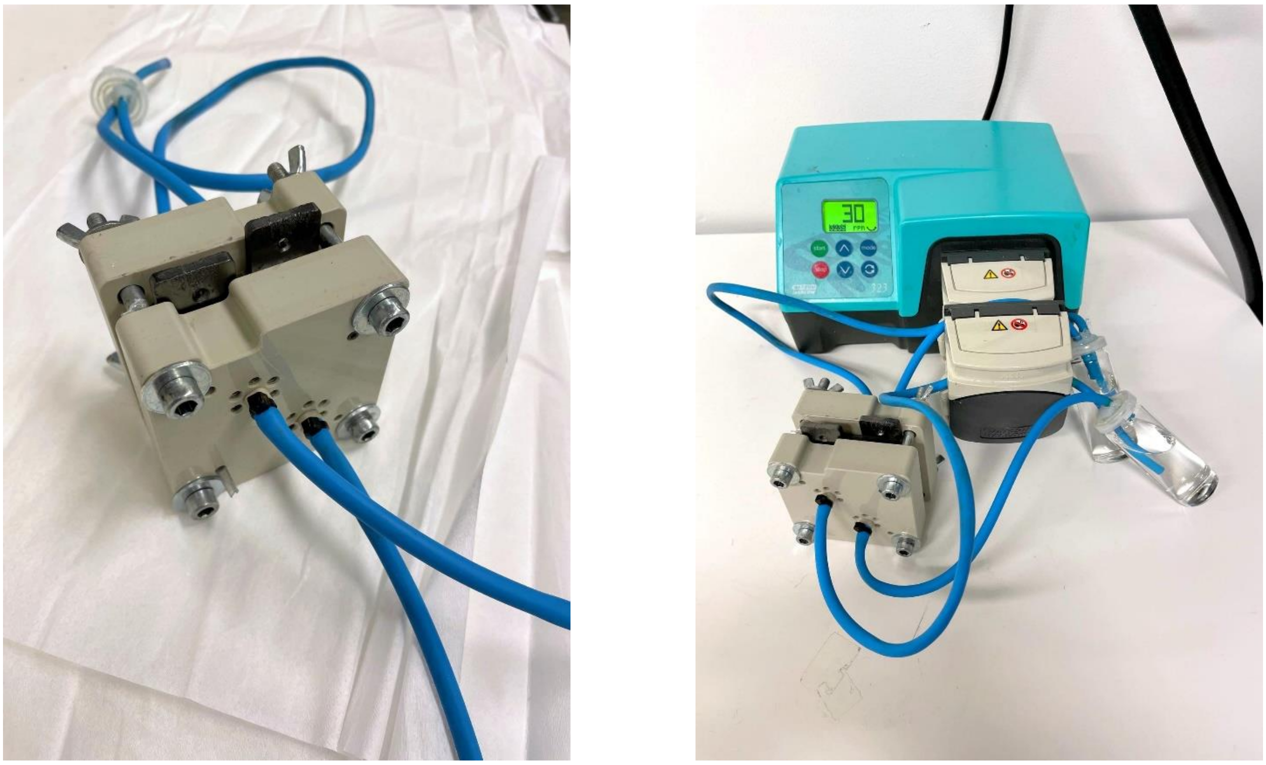

Figure S1. left) assembled cell made of Polypropylene right) leakage test before taking to the glovebox

Membrane testing

In an effort to identify cost-effective alternatives to the expensive Nafion membrane, our study explored a range of membranes to effectively separate solutions in a controlled environment. These alternatives included a hydrophobic polypropylene membrane filter with a 1.2 µm pore size, a hydrophilic PTFE membrane filter with a 0.22 µm pore size, a hydrophilic polypropylene membrane filter with a 0.2 µm pore size, along with high and low EEO agarose gel membranes, all operated under low pump flow rates. Despite these varied approaches, our experiments consistently demonstrated that the membranes were unable to prevent the solutions from mixing. Moreover, attempts to employ dual membrane systems resulted in suboptimal outcomes, where the solutions either mixed or the system encountered significantly increased resistance, highlighting the challenges in finding a viable alternative to the Nafion membrane in such applications.

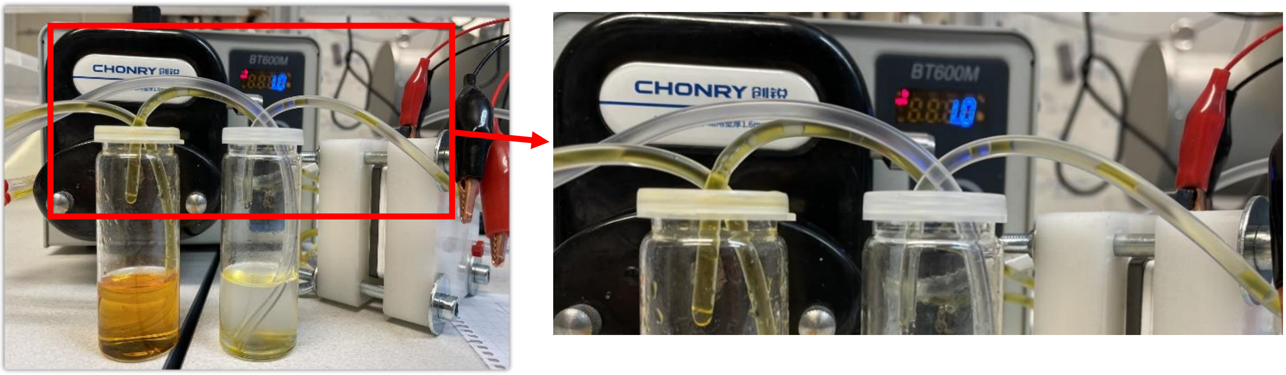

Figure S2. Testing Ferrocene in Pc solution against water using High EEO agarose gel membrane, showing the solutions getting mix after couple of hours

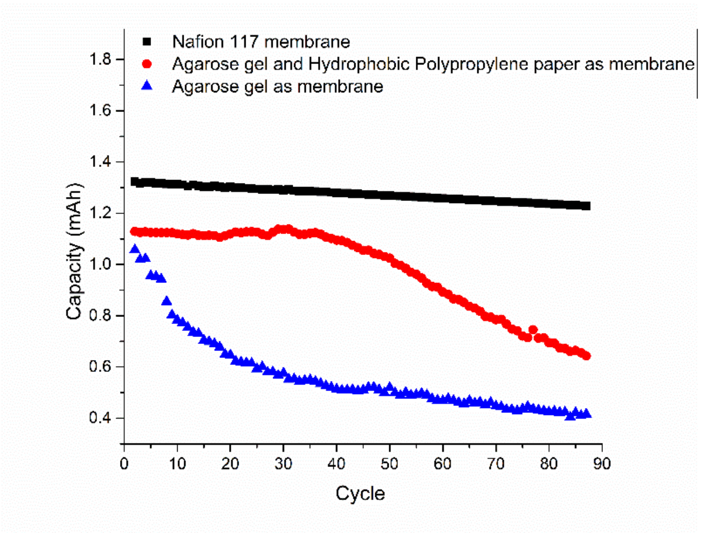

Figure S3- Comparison of different membranes in the same batteries, 1mM DMFc + 10 mM LiTB in Pc as Negolyte and 30 mM KFCN(II) /30 mM KFCN (III) + 100 mM LiCl in water

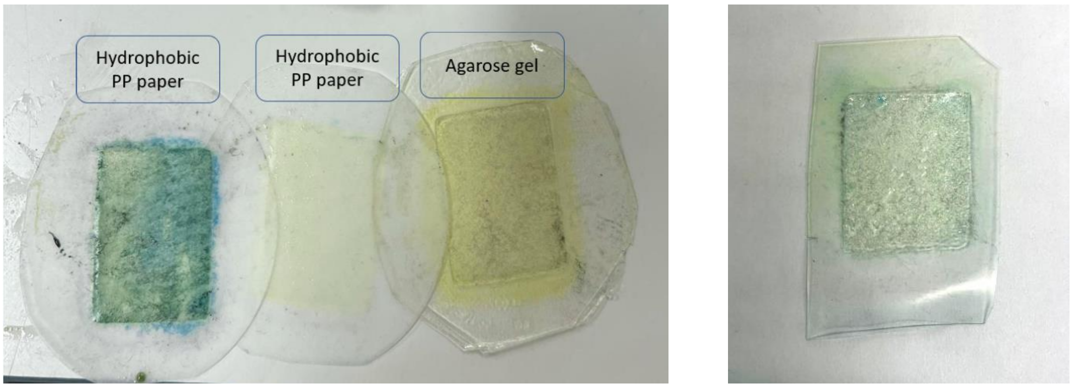

Figure S4. Left) set of hydrophobic papers and agarose gel as membrane Right) Nafion N-117 as membrane after the battery tests

Battery testing

Table S1 provides a detailed overview of the assembled batteries, highlighting varying volumes and concentrations investigated to substantiate the operational impact of the Galvani potential difference across different conditions.

Table S1-Assembled Battery details

Anolyte (organic)	Posolyte (water)	Supporting electrolyte in organic phase	Supporting electrolyte in water phase
DMFc in TFT (2.5 mM, 10 mL)	KFCN (II) (30 mM) and KFCN (III) (30 mM), 20 mL	LiTB (10 mM)	LiCl (100 mM)
DMFc in DCE (2.5 mM, 10 mL)	20 mL	LiTB (10 mM)	LiCl (100 mM)
DMFc in PC (1 mM, 10 mL)	20 mL	LiTB (10 mM)	LiCl (100 mM)
Fc in TFT (5 mM, 17.5 mL)	25 mL	LiTB (10 mM)	LiCl (100 mM)
Fc in DCE (5 mM, 12.5 mL)	25 mL	LiTB (10 mM)	LiCl (100 mM)
Fc in PC (2.5 mM, 15 mL)	25 mL	LiTB (10 mM)	LiCl (100 mM)

Estimation of the standard potentials of Decamethylferrocene and Ferrocene in Polypropylene Carbonate

In this study, an internal reference electrode consisting of Ag/Ag^+ reference (10 mM $AgNO_3$ in acetonitrile) was employed to ascertain the potential difference between ferrocene (Fc) and decamethylferrocene (DMFc) in propylene carbonate (PC).

Potential of Ferrocene in PC *vs* Li^+/Li = 3.376 V[S1]

Potential of Li^+/Li in PC *vs* SHE = –2.790 V[S2]

Potential of Fc in PC *vs* SHE = 0.576 V

potential difference between DMFc and Fc in PC = 0.521 V

Potential of DMFc in PC *vs* SHE = 0.055 V

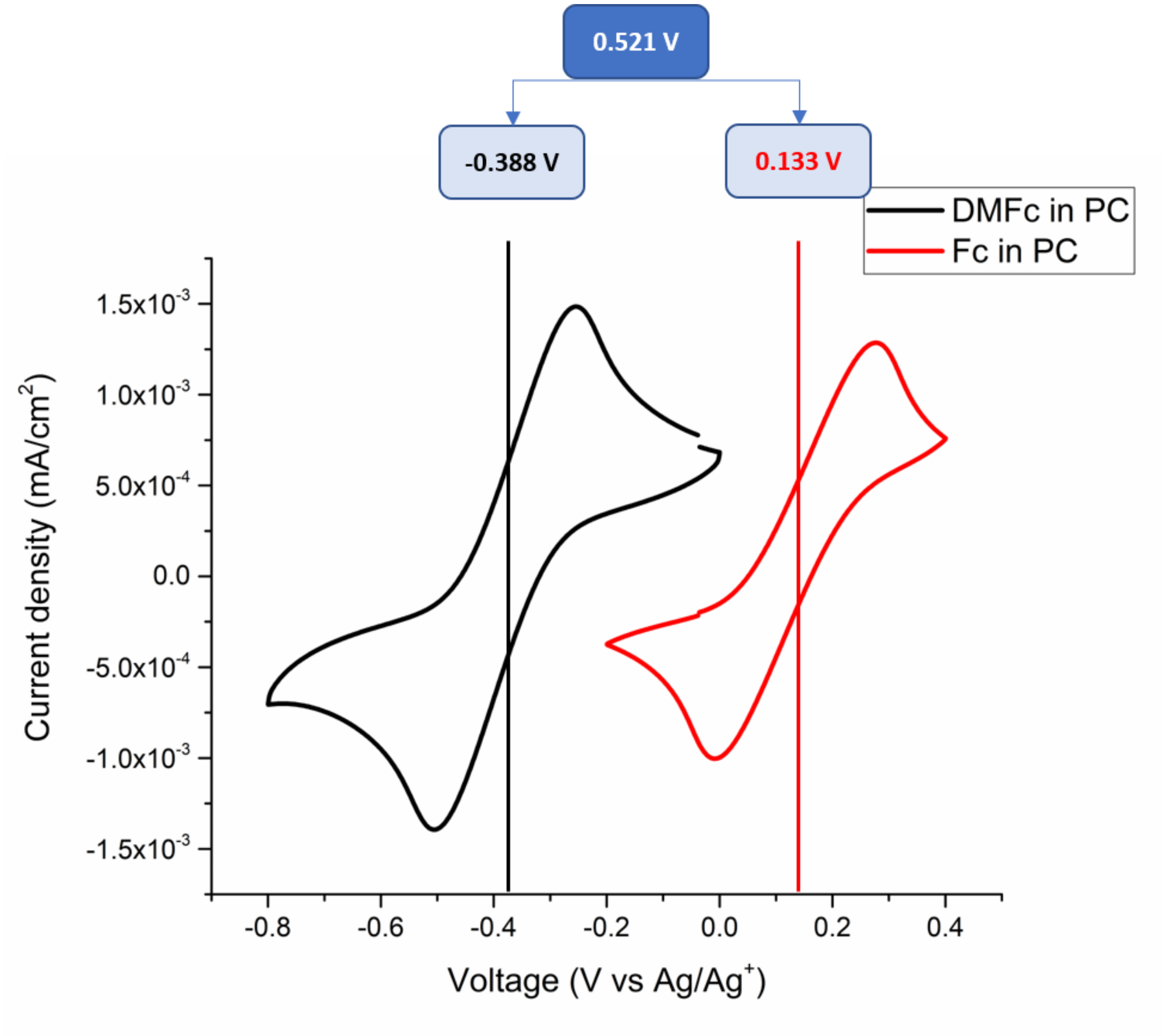

Figure S5. Potential difference between FC and DMFc in PC

Reactions in the cell

The increased voltage can be understood if the whole reaction mechanism is considered. Upon charge, $[Fe(II)(CN)_6]^{4-}$ is oxidized to $[Fe(III)(CN)_6]^{3-}$ in the aqueous phase, and ferrocenium Fc^+ or $DMFc^+$ is reduced to Fc or DMFc. But to keep the phases electroneutral, the overall reaction requires a transfer of Li^+ from organic phase to aqueous phase.

During charge

$$[\mathrm{Fe(CN)_6}]^{4-}(\mathrm{aq}) \rightarrow [\mathrm{Fe(CN)_6}]^{3-}(\mathrm{aq}) + e^- \quad \text{(S1)}$$

$$\mathrm{Fc}^+(o) + e^- \rightarrow \mathrm{Fc}(o) \quad \text{(S2)}$$

$$\mathrm{Li}^{+}(\mathrm{aq}) \rightarrow \mathrm{Li}^{+}(o) \tag{S3}$$

The total reaction is:

$$[\mathrm{Fe(CN)_6}]^{4-}(\mathrm{aq}) + \mathrm{Fc}^{+}(o) + \mathrm{Li}^{+}(aq) \rightarrow [\mathrm{Fe(CN)_6}]^{3-}(\mathrm{aq}) + \mathrm{Fc}(o) + \mathrm{Li}^{+}(\mathrm{o}) \tag{S4}$$

The Gibbs energy change for this reaction can be written as

$$\Delta G = F\left(E_{[\mathrm{Fe(CN)_6}]^{4-}/[\mathrm{Fe(CN)_6}]^{3-}} - E_{\mathrm{Fc/Fc^+}}\right) + \Delta G^{\mathrm{w}\rightarrow\mathrm{o}}_{\mathrm{tr,Li^+}} \tag{S5}$$

Eq. S5 illustrates that the Gibbs energy of the reaction is composed of the half-cell reactions and the energy to transfer Li^+ from water to oil. With TFT and DCE the standard transfer energy of Li^+ from water to oil is 77.7 kJ/mol or 55.8 kJ/mol (see next section), so significantly more energy is required in the charging phase. But upon discharge the same amount of energy is recovered. With PC the transfer energies are close to 0, so the reaction above actually becomes spontaneous if Fc is used as the redox couple in PC phase.

Thermodynamics of the ITIES and partitioning of the species

According to the electrochemistry textbooks[S3,S4] Galvani potential of the phase ϕ is defined as the sum of the outer potential ψ and inner potential χ, $\phi = \chi + \psi$ and it is included in the expression of the electrochemical potential of the species $\tilde{\mu}_i = \mu_i + z_i F \phi_i$, where μ_i is the chemical potential of the species i, F is the Faraday constant, and z_i is the charge of the species i. Here, the chemical potential describes the energy of adding a species i to the phase, and $z_i F\psi$ is the electrostatic work to bring one mol of species i from vacuum to the surface of the phase and $z_i F\chi$ is the work to cross the interface of the phase with vacuum. Chemical potential is connected to the Gibbs free energy G by

$$\mu_i = \left(\frac{\partial G}{\partial N_i}\right)_{T,p,N_{j\neq i}} \tag{S6}$$

where N is the number of particles. If temperature T and pressure p are constant, change in the Gibbs free energy is

$$dG = \sum_{i=1}^{n} \mu_i dN_i \tag{S7}$$

When species i is in equilibrium in two phases, electrochemical potential of the species i is equal. This section follows the description of refs. [S3] and [S5-S7].

$$\tilde{\mu}_i^w = \tilde{\mu}_i^o \tag{S8}$$

$$\mu_i^w + z_i F \phi_i^w = \mu_i^o + z_i F \phi_i^o \tag{S9}$$

$$\mu_i^{0,\mathrm{w}} + RT\ln a_i^w + z_i F \phi_i^w = \mu_i^{0,\mathrm{o}} + RT\ln a_i^o + z_i F \phi_i^o \tag{S10}$$

$$\phi_i^w - \phi_i^o = \frac{\mu_i^{0,\mathrm{o}} - \mu_i^{0,\mathrm{w}}}{z_i F} + \frac{RT}{z_i F} ln \frac{a_i^o}{a_i^w} \tag{S11}$$

Here, we can define the Galvani potential difference between aqueous and organic phases as $\Delta_o^w\phi = \phi_i^w - \phi_i^o$ and the standard Gibbs energy of transfer and standard transfer potential can be defined as $\Delta G_{\mathrm{tr},i}^{0,w\to o} = \mu_i^{0,\mathrm{o}} - \mu_i^{0,\mathrm{w}}$ resulting in the definition of the standard potential of ion transfer as:

$$\Delta_o^w\phi_i^0 = \frac{\Delta G_{\mathrm{tr},i}^{0,w\to o}}{z_iF} = \frac{\mu_i^{0,\mathrm{o}} - \mu_i^{0,\mathrm{w}}}{z_iF} \qquad \text{(S12)}$$

where $\mu_i^{0,\alpha}$ is the standard chemical potential of *i* in either phase (α = o or w), z_i is the charge of *i*, and *F* is the Faraday constant. Equation S11 illustrates the connection between the Galvani potential difference and partition of ions, resulting in the Nernst equation for ion partition (analogous with metal electrodes):

$$\phi^w - \phi^o = \Delta_o^w\phi = \Delta_o^w\phi_i^0 + \frac{RT}{z_iF}ln\frac{a_i^o}{a_i^w} = \Delta_o^w\phi_i^{0'} + \frac{RT}{z_iF}ln\frac{c_i^o}{c_i^w} \qquad \text{(S13)}$$

where $\Delta_o^w\phi_i^{0'}$ is the formal potential of ion transfer for species *i* from water to oil, *R* is the molar gas constant, *T* is the temperature and a_i and c_i are the activity and concentration of species *i*, respectively.

The determination of a transfer potential of an ion requires an extra thermodynamic assumption. The most commonly used is the TATB hypothesis, which states that the transfer energy of tetraphenyl borate and tetraphenylarsenium are equal between any pair of solvents[S8] (*i.e.* by choosing that $\Delta_o^w\phi_{\mathrm{TPAs}^+}^0 = -\Delta_o^w\phi_{\mathrm{TPB}^-}^0$ independent of the solvent). This assumption is partly justified as the size of the molecule is not affected greatly by the centre atom and the charge is shielded by the bulky phenyl rings. The $\Delta G_{\mathrm{tr},\,i}^{w\to o}$ values depend on the organic solvent, but it has been observed experimentally that there is a linear correlation between the $\Delta G_{\mathrm{tr},\,i}^{w\to o}$ values determined for different solvents. Hence this correlation can be used to estimate transfer energies when experimental data is not available. [S9-S12] This correlation between DCE and TFT is illustrated in Fig. S6, with the data for transfer energies taken from refs. S11-S15.

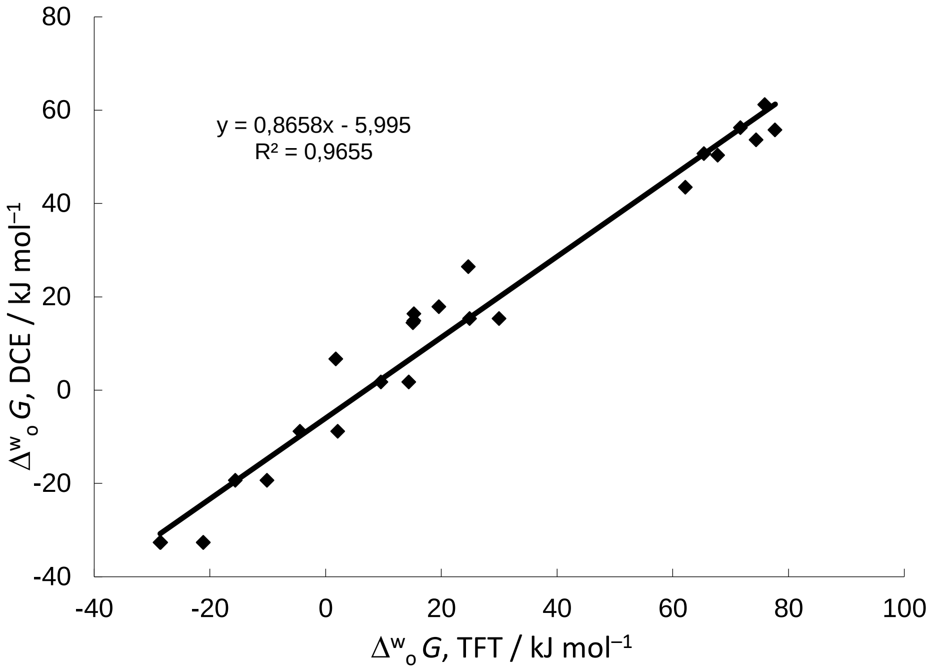

Figure S6. Experimental correlation of transfer energies of different ions between water and TFT and water and DCE, with the data for transfer energies taken from refs. S11-S15.

Equation S13 has two significant meanings: the distribution of species can be controlled by controlling the Galvani potential difference across the interface, and the Galvani potential difference can be adjusted by the distribution of species between the phases. In a system where ionic species of the two immiscible liquid phases are in equilibrium, the equilibrium potential difference across the interface (Eq. S2) has to be fulfilled for all the species. This can be calculated if the initial amounts and the transfer energies are known, by taking into account mass balance equations.[S16] The mass balance for species i is

$$n_{i,\,\mathrm{total}} = n_i^o + n_i^w \qquad \text{(S14)}$$

$$V^o c_{i,\,\mathrm{initial}}^o + V^w c_{i,\mathrm{initial}}^w = V^o c_i^o + V^w c_i^w \qquad \text{(S15)}$$

Additionally, the electroneutrality condition of both phases must be fulfilled:

$$\sum_i z_i c_i^w = \sum_i z_i c_i^o = 0 \qquad \text{(S16)}$$

If we assume that ratio of all the activity coefficients between aqueous and oil phases ≈ 1, combination of Equations S13-S16 results

$$\sum_i z_i \frac{V^o c_{i,\text{ initial}}^o + V^w c_{i,\text{initial}}^w}{V^w + V^o \, exp\left[\frac{zF}{RT}(\Delta_o^w \varphi - \Delta_o^w \varphi_i^0)\right]} = 0 \qquad \text{(S17)}$$

Equation S17 can be solved numerically to evaluate the Galvani potential difference of the system in equilibrium, and Nernst equation and mass balance equations can be used to calculate the compositions of both phases. The results in the case of 10 mL of 10 mM LiTB in the organic phase and 10 mL of 100 mM LiCl in the aqueous phase are shown in Tables S2 and S3 for DCE and TFT. The transfer energies for Li^+ and Cl^- were taken from ref. [S14] as 55.8 and 50.9 kJ/mol for DCE and 77.7 and 65.6 kJ/mol for TFT, and transfer energy of TB^- was taken as –68.5 kJ/mol for transfer from water to DCE[S17] and evaluated as –72.2 kJ/mol for transfer from water to TFT based on the linear correlation in Fig. S6.

Table S2- TFT: $\Delta_o^w\phi_{eq}$ = 0.734 V

	$\Delta_o^w\phi^0$, V	c^0_w, mM	c^0_o, mM	c_w, mM	c_o, mM
Li^+	0.805	100	10	103.6	6.4
TB^-	0.748	-	10	3.6	6,4
Cl^-	–0.678	100	-	100	0

Table S3- DCE: $\Delta_o^w\phi_{eq}$ = 0.519 V

	$\Delta_o^w\phi^0$, V	c^0_w, mM	c^0_o, mM	c_w, mM	c_o, mM
Li^+	0.578	100	10	100.01	9.99
TB^-	0.71	-	10	0.01	9.99
Cl^-	–0.530	100	-	100	0

Let us next consider the solubility of participating reactants. Here the trick is that the partition of the ionic species is controlled by the Galvani potential difference.[S5-S7] Partition coefficient is defined as the activity of species *i* in oil divided the activity in water:

$$P = \frac{a_i^{\text{o}}}{a_i^{\text{w}}} \qquad \text{(S18)}$$

From Eq. S13 we can reformulate

$$P = \frac{a_i^{\text{o}}}{a_i^{\text{w}}} = \exp\left[\frac{z_i F}{RT}\left(\Delta_{\text{o}}^{\text{w}}\phi - \Delta_{\text{o}}^{\text{w}}\phi_i^0\right)\right] \qquad \text{(S19)}$$

Ferri/ferrocyanide transfer potential is less than –0.68 V for TFT and less than –0.53 V for DCE as transfer of neither is visible before transfer of chloride, so log *P* for ferri/ferrocyanide are less than –102 and –76 in TFT and –76 and –57 in DCE at the given Galvani potential differences. This illustrated that almost no ferri/ferrocyanide will partition in the aqueous phase. TFT-water partition coefficient of Fc has been estimated as 13 400 (log *P* = 4.13) and the standard ion transfer potential of Fc^{+} is 0.115 V for TFT-water interface.[S18] Partition coefficient can be calculated from equation above, resulting in log *P* of 10.48. The values have not been evaluated for DMFc, but the molecule is much more hydrophobic so the partition coefficient will be even higher.

Battery experiments

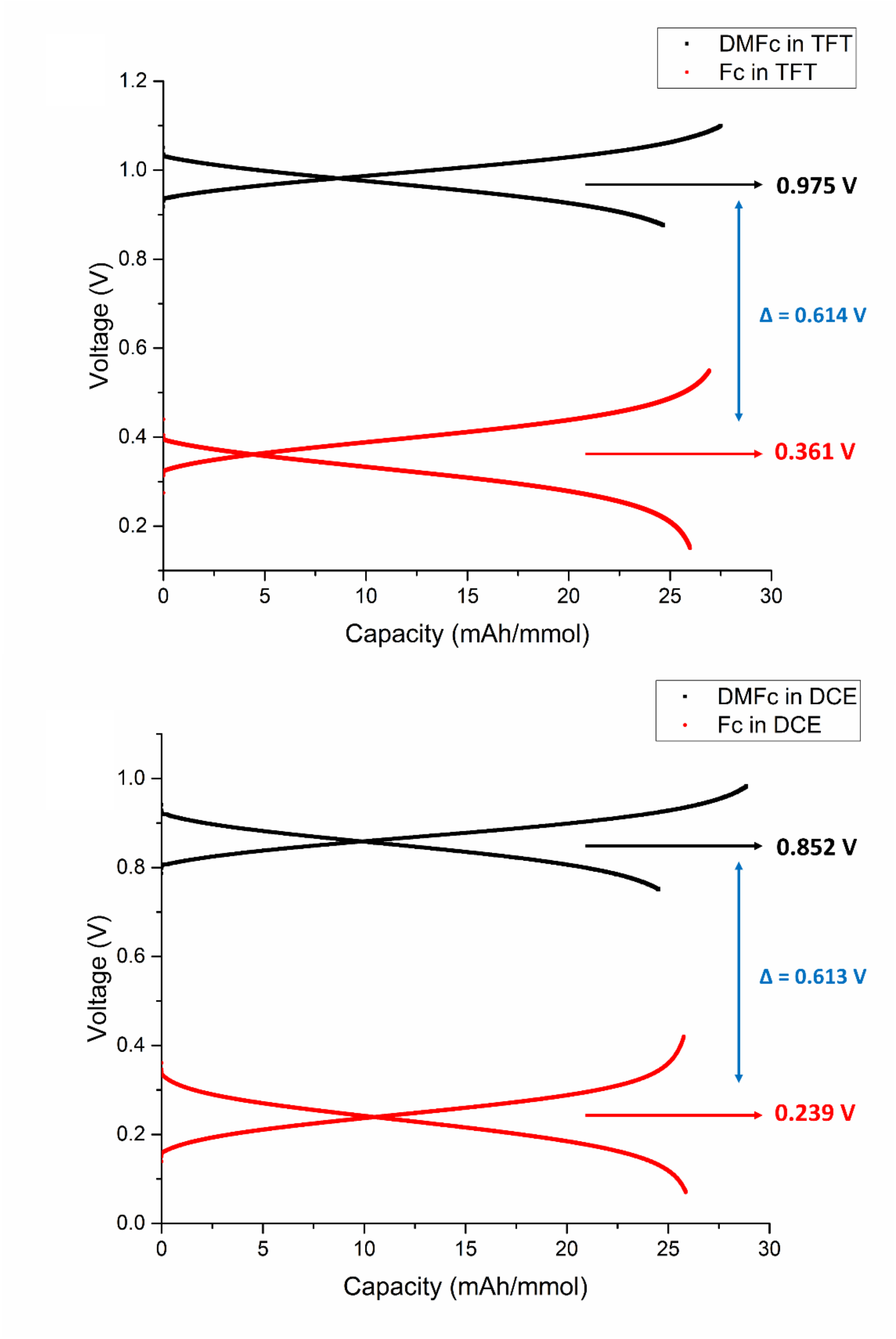

Figure S7- Cell potentials obtained with different redox couples in top) Trifluorotoluene (TFT) and bottom) Dichloroethane (DCE). The average cell voltage at 50% state of charge as well as the potential difference between Ferocene (Fc) and Decamethylferrocene (DMFc) are highlighted.

Table S4- Comparison between the theoretical and measured potentials of assembled batteries using the same posolyte (30 mM KFCN(II) /30 mM KFCN (III) + 100 mM LiCl in water)

Anolyte	Theoretical potential (V)	Measured potential (V)	Difference (V)	Difference considering activity (V)
DMFc in TFT	1.121	0.975	0.146	0.101
DMFc in DCE	0.916	0.852	0.064	0.027
DMFc in PC	0.412	0.405	0.007	0.010
Fc in TFT	0.481	0.361	0.120	0.076
Fc in DCE	0.346	0.239	0.107	0.070
Fc in PC	–0.109	–0.160	0.051	0.054

We can also consider the effects of activities on the measured voltages. From Eq. S13 the effect of the activity coefficients is evident:

$$\Delta_o^w \phi = \Delta_o^w \phi_i^0 + \frac{RT}{z_i F} \ln \frac{a_i^o}{a_i^w} = \Delta_o^w \phi_i^0 + \frac{RT}{z_i F} ln \frac{c_i^o \gamma_i^o}{c_i^w \gamma_i^w} = \Delta_o^w \phi_i^0 + \frac{RT}{z_i F} \ln \frac{\gamma_i^o}{\gamma_i^w} + \frac{RT}{z_i F} \ln \frac{c_i^o}{c_i^w} \quad \text{(S20)}$$

For evaluation of the activities, extended Debye-Hückel equation can be utilized:

$$log_{10}\gamma = \frac{-z_{Li^+}^2 A\sqrt{I}}{1+ B' a\sqrt{I}} \quad \text{(S21)}$$

where the ionic strength is

$$I = \frac{1}{2}\sum_i c_i z_i^2 \quad \text{(S22)}$$

and A and B are constants depending on relative permittivity of the solvent and a is the effective diameter of the ion. For TFT, DCE and PC, A is 12.70, 10.54, and 0.69 and B is 9.60, 9.02 and 3.64, respectively (relative permittivities are 9.2, 10.4 and 64). The ionic strength for the organic solvent was considered as

$$I_{Organic} = \frac{1}{2}[(0.01)_{Li^+}(1)^2 + (0.01)_{TB^-}(-1)^2] = \mathbf{0.01\ M} \quad \text{(S23)}$$

If we consider that lithium is hydrated in the organic solvent with radius of 2.5 Å, the activity coefficients can be calculated as 0.14, 0.19 and 0.87 for TFT, DCE and PC, while the activity of 100 mM LiCl in aqueous solution is 0.78 based on Eq. S21. Effect of the activity coefficients would

therefore be –45, –37 and 3 mV for TFT, DCE and PC. This correction improves the agreement between the expected and measured voltages.

Cyclability and coulombic efficiency of batteries

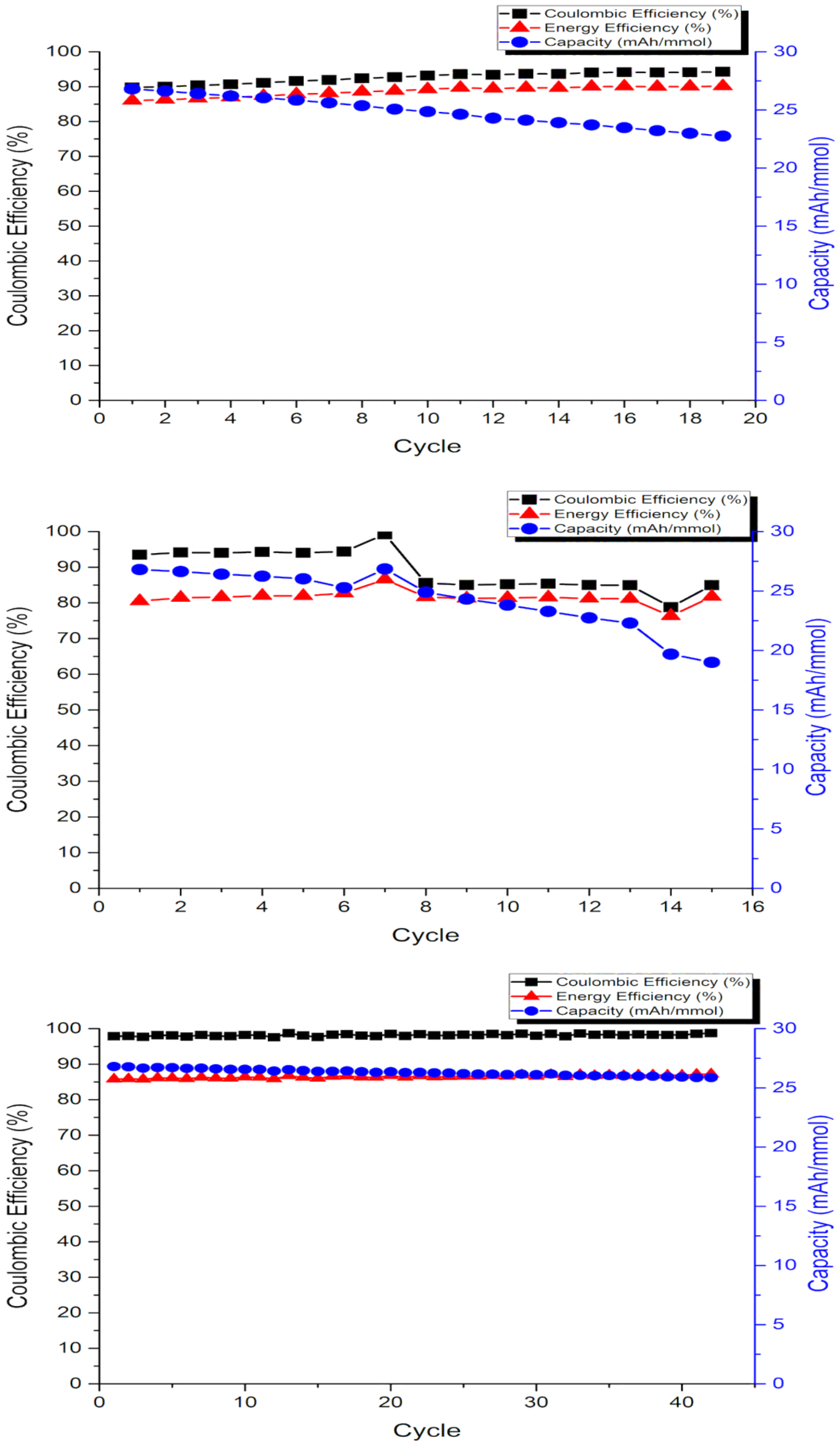

Figure S8- Cycle performance of batteries DMFC in top) TFT, middle) DCE, and bottom) PC as negolyte vs KFCN in water as posolyte

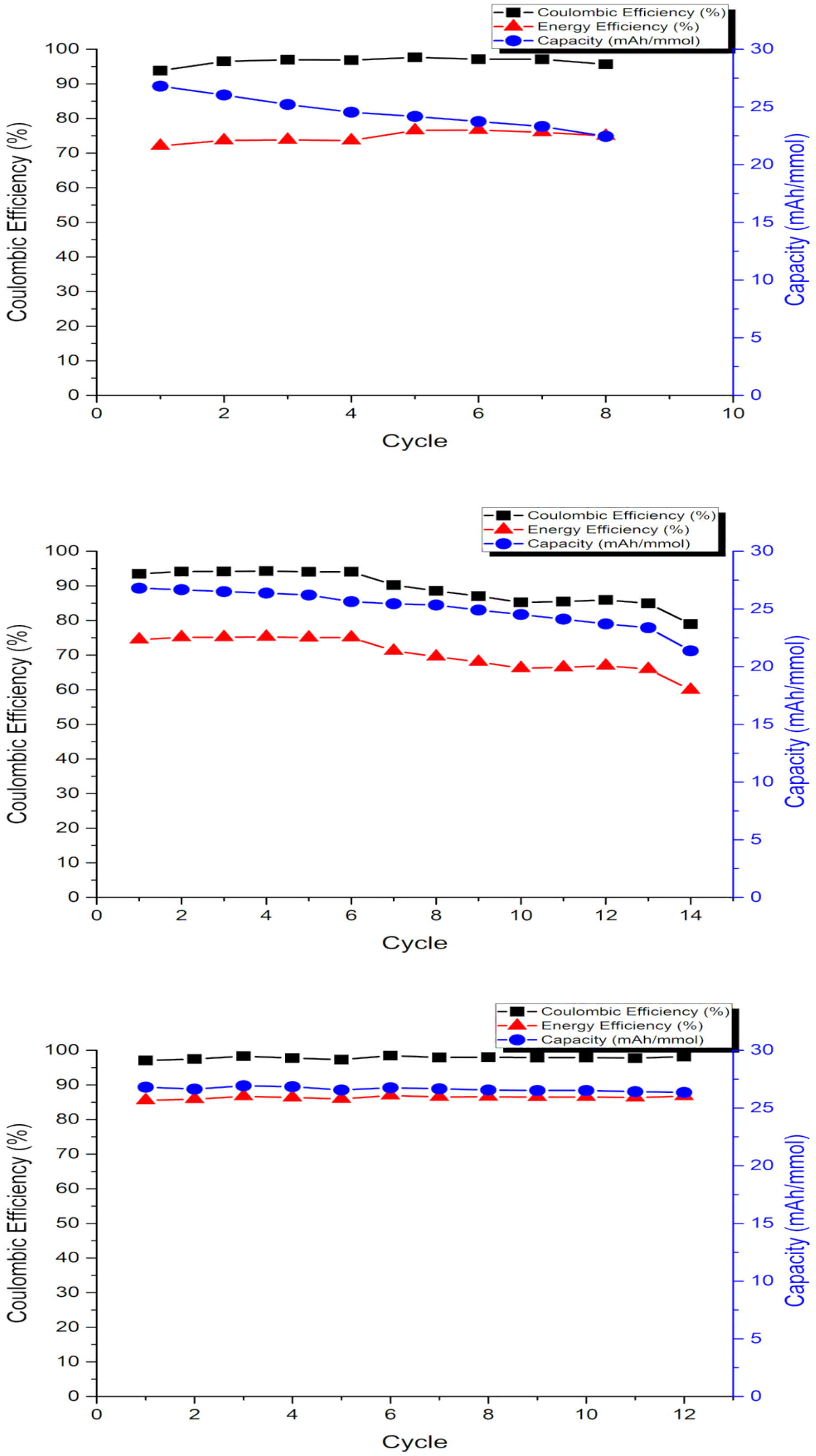

Figure S9-Cycling behaviour of different batteries FC in top) TFT, middle) DCE, and bottom) PC as negolyte vs KFCN in water as posolyte

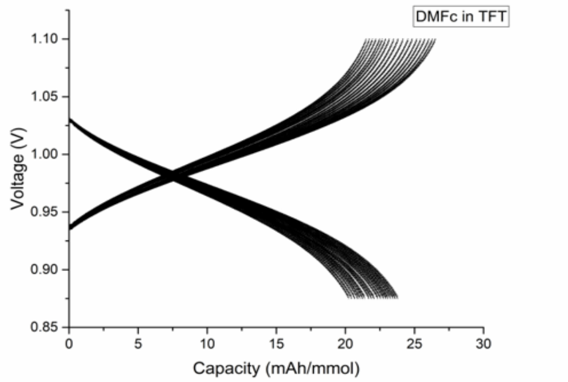
DMFc in TFT
Voltage (V)
Capacity (mAh/mmol)

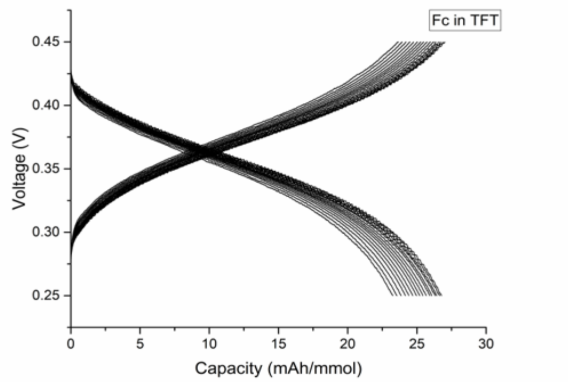
Fc in TFT
Voltage (V)
Capacity (mAh/mmol)

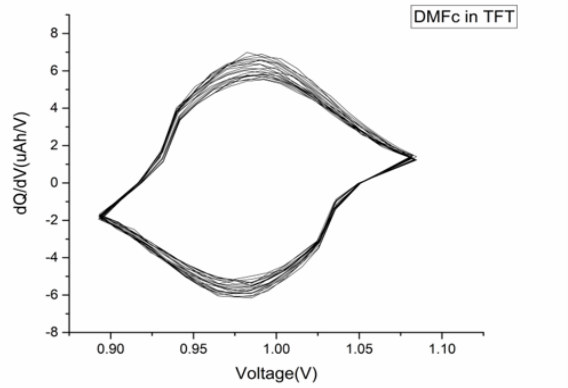
DMFc in TFT
dQ/dV(uAh/V)
Voltage(V)

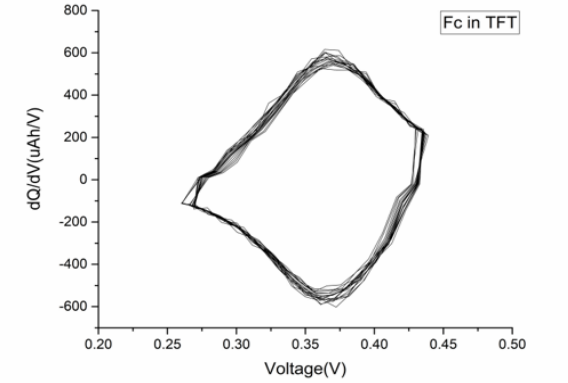
Fc in TFT
dQ/dV(uAh/V)
Voltage(V)

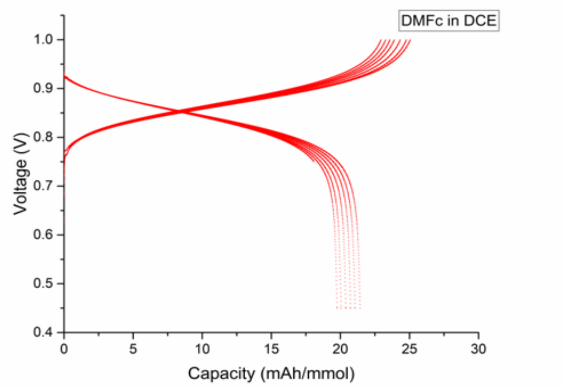
DMFc in DCE
Voltage (V)
Capacity (mAh/mmol)

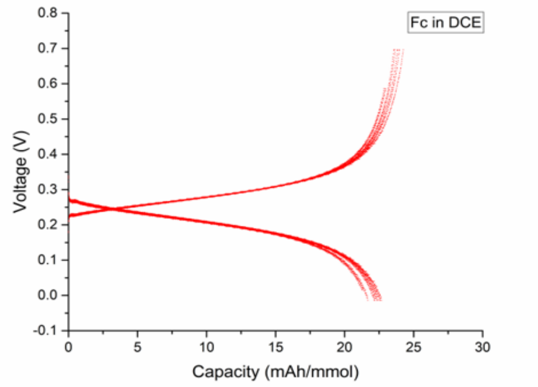
Fc in DCE
Voltage (V)
Capacity (mAh/mmol)

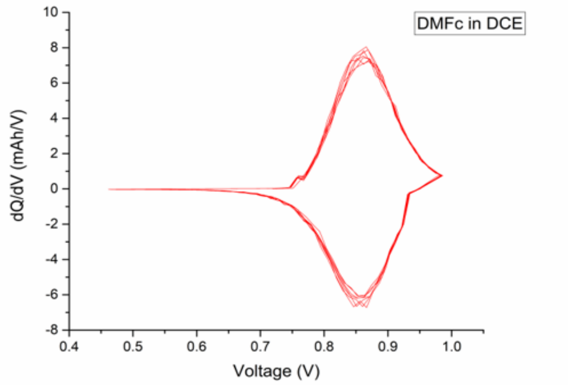
DMFc in DCE
dQ/dV (mAh/V)
Voltage (V)

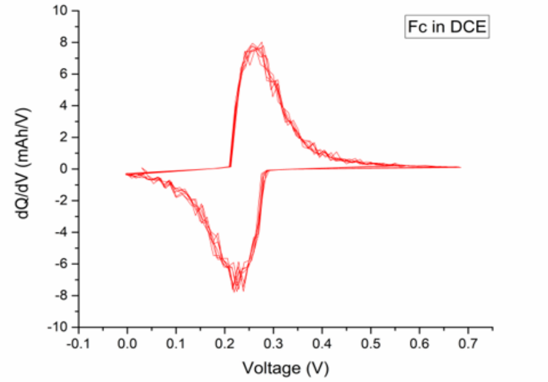
Fc in DCE
dQ/dV (mAh/V)
Voltage (V)

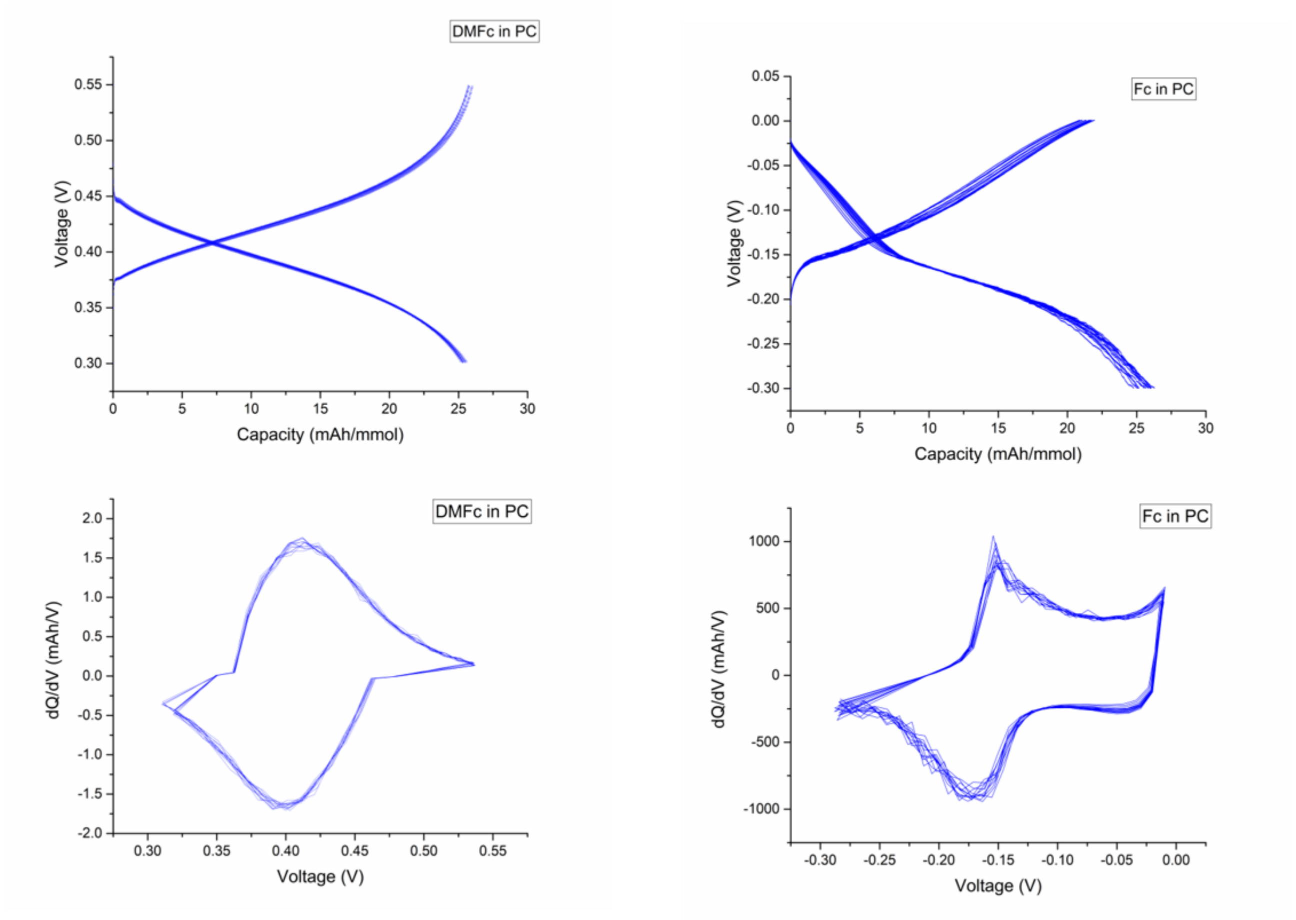

Figure S10- Cycle performance of batteries and dQ/dV values. Negolyte side of the battery mentioned, Posolyte is the same for all: 30 mM KFCN (II) + KFCN (III) + 100mM LiCl in water

Figs. S8-S10 illustrate that, during cycling, the potential of batteries remains constant, while the capacity decreases. The observed capacity fading is more pronounced compared to water-based flow batteries, attributed to the lower boiling point of the employed organic solvents.

Table S5-Resistance and capacity fade of anolytes

**Anolyte**	**Boiling Point (ºC)**	**Capacity fade (per cycle)**	**Resistance from *IR* drop (ohm)**	**Current (mA/cm^2)**	***IR* drop (V)**
DMFc in TFT	102	0.0053 %	75	0.2	0.075
DMFc in DCE	83	0.0125 %	145	0.2	0.145
DMFc in PC	242	0.0002 %	27	0.08	0.010
Fc in TFT	102	0.0200 %	150	0.08	0.060
Fc in DCE	83	0.0221 %	70	0.08	0.028
Fc in PC	242	0.0011 %	75	0.08	0.030

Cyclic Voltammograms

Cyclic voltammetry has been done to confirm both DMFc and Fc are stable in the tested solvents.

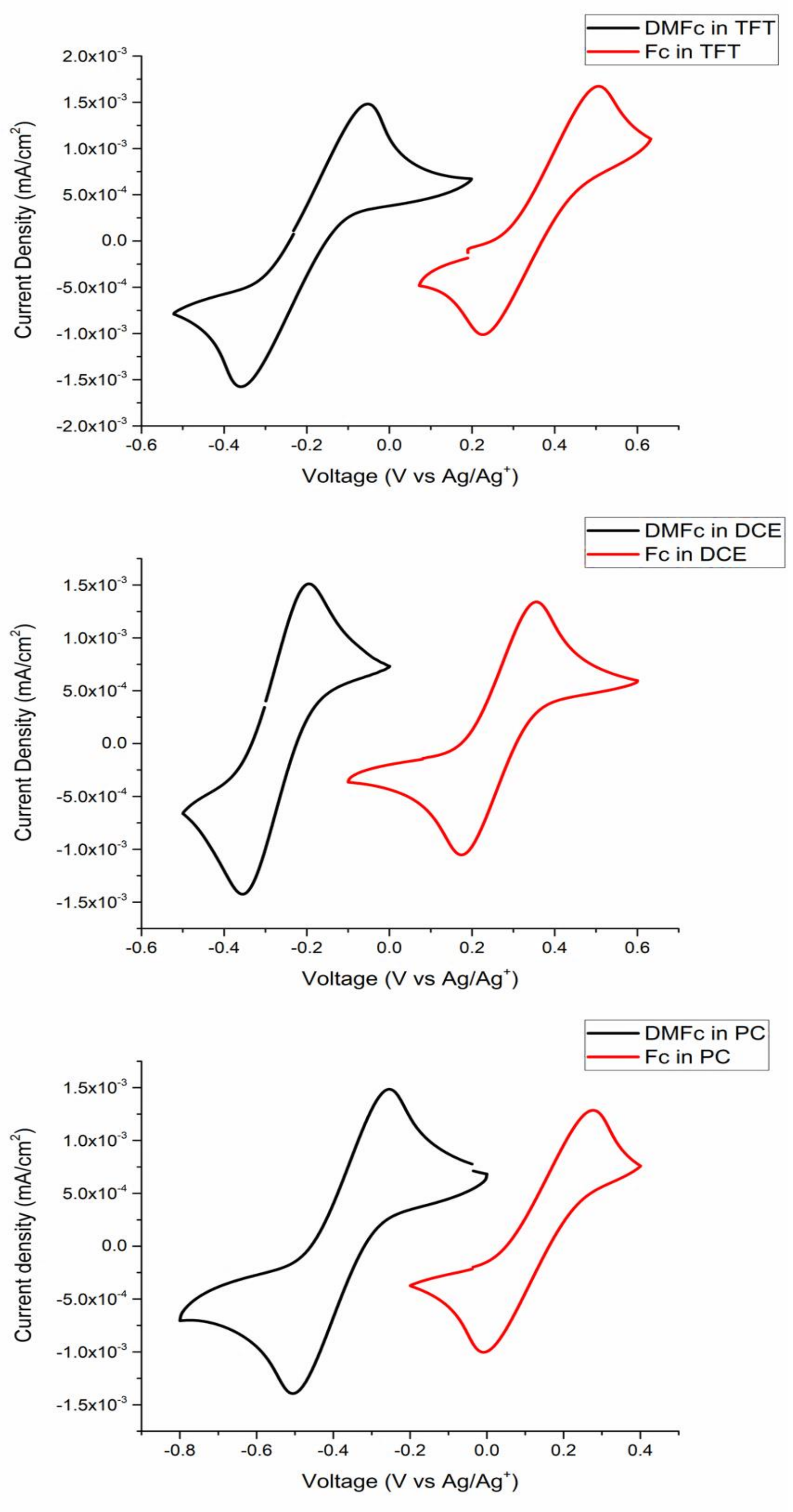

Figure S11- Cyclic Voltammograms of Fc and DMFc in TFT, DCE and PC solvents vs Ag/Ag$^+$ reference (10 mM $AgNO_3$ in acetonitrile) as internal reference electrode

Impedance spectra

EIS spectra have been measured to characterize the ohmic resistances (R_{ohm}) defined in the high-frequency region. R_{ohm} encompasses contact resistances between the electrolyte and electrodes, electrolyte resistances, and membrane resistance within the cell. For instance, the ohmic resistance of the system shown in Figure S12 is 2.4 Ω which is around 10 times higher than a vanadium flow battery (aqueous)[S19]. As the impedance spectra for such a system include capacitance and Faradaic impedance of the positive and negative electrodes as well as the interface, the equivalent circuit can be generalized as shown in Figure S13. Since it is very difficult to distinguish the impedance from each part, we have used the current step instead to evaluate the cell resistance. However, the resistance taken from *IR* drop has faradic current with itself but it is easier to measure and also the detailed analysis of the EIS spectra and resistance is outside the scope of the paper.

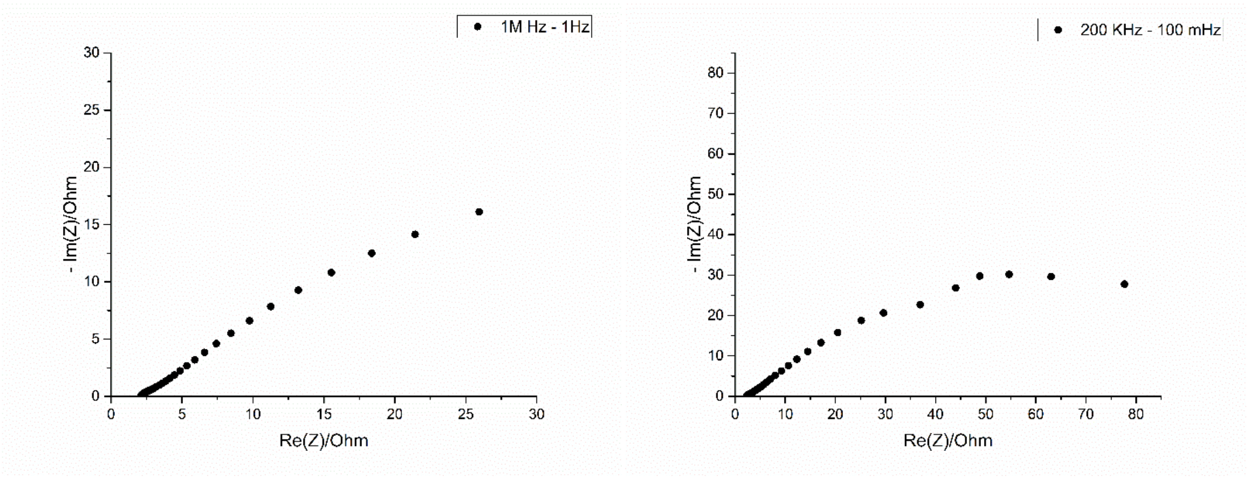

Figure S12- EIS from Battery - 1mM Ferrocene + 10 mM LiTB in DCE solvent as negolyte and 30 mM KFCN (II) + KFCN (III) + 100mM LiCl in water as posolyte at different frequency ranges at state of charge close to 100%, measured at the open circuit potential.

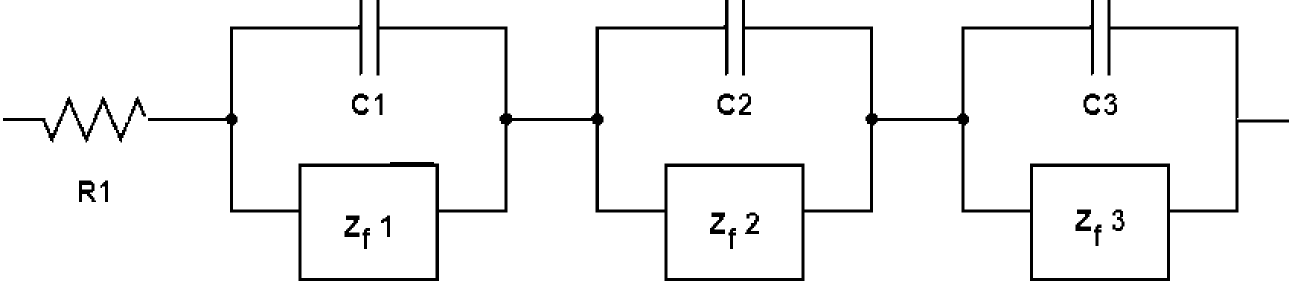

Figure S13- Equivalent circuit of the system, where R1 is the sum of all the ohmic resistances in the system, and capacitors C1 to C3 refer to double layer capacitances of the negative, positive electrode as well as the liquid-liquid interface. Faradaic reactions at the positive and negative electrodes as well as at the liquid-liquid interface can be generalized by Faradaic impedances Z_f1-3. Here, these impedances include charge transfer resistance of a porous electrode as well as mass transfer.

Supplementary References

S1 E. C. Cengiz, J. Rizell, M. Sadd, A. Matic and N. Mozhzhukhina, *J. Electrochem. Soc.*, 2021, **168**, 120539.

S2 N. Mozhzhukhina and E. J. Calvo, *J. Electrochem. Soc.*, 2017, **164**, A2295–A2297.

S3 H. H. Girault, *Analytical and Physical Electrochemistry*, EPFL Press: Lausanne, **2004.**

S4 A. J. Bard and L. R. Faulkner, *Electrochemical methods: fundamentals and applications*, John Wiley & Sons, 2nd edition., 2001.

S5 P. Peljo, H.H. Girault, Liquid/Liquid Interfaces, Electrochemistry at. In *Encyclopedia of Analytical Chemistry*; John Wiley & Sons, Ltd.: Chichester, U.K., 2012

S6 H. H. Girault, D. Schiffrin, Electrochemistry of Liquid-Liquid Interfaces. in *Electroanalytical Chemistry*, CRC Press, 1st Edition., 1988.

S7 Z. Samec, Pure Appl. Chem. 2004, 76, 2147– 2180.

S8 A. J. Parker, *Electrochemica Acta*, 1976, **21**, 671–679.

S9 S. Wilke, T. Zerihun, *J. Electroanal. Chem*., 2001, **515**, 52-60.

S10 B. Hundhammer, C. Müller, T. Solomon, H. Alemu, H. Hassen, *J. Electroanal. Chem*., 1991, **319** , 125-135.

S11 J. Liu, X. Zheng, Y. Hua, J. Deng, P. He, Z. Yu, X. Zhang, X. Shi and Y. Shao, *ChemElectroChem*, 2022, **9**, 13.

S12 E. Smirnov, P. Peljo, M. D. Scanlon and H. H. Girault, *Electrochim Acta*, 2016, **197**, 362–373.

S13 A. J. Olaya, P. Ge, H. H. Girault, *Electrochem. Commun*, 2012, **19**, 101–104.

S14 A. Trojánek, V. Mareček, J. Langmaier and Z. Samec, *Electrochim Acta*, 2023, **449**, 142222.

S15 A. Trojánek, V. Mareček, J. Fiedler and Z. Samec, *Electrochim Acta*, 2023, **465**, 142966

S16 T. Kakiuchi, in: A. Volkov, G., D.W. Deamer (Eds), *Liquid-liquid interfaces, Theory and Methods*, CRC Press, Boca Raton, 1996, pp. 1-18.

S17 A. J. Olaya, M. A. Méndez, F. Cortes-Salazar and H. H. Girault, *Journal of Electroanalytical Chemistry*, 2010, **644**, 60–66.

S18 P. Peljo, E. Smirnov and H. H. Girault, *J. Electroanal. Chem.*, 2016, **779**, 187–198.

S19 C. Wan, R. Jacquemond, M. Chiang, K. Nijmeijer, F. Brushett and A. Forner-Cuenca, *Advanced Materials*, 2021, **33**, 16.
